# Evaluation of Photocatalytic and Protein Adsorption Properties of Anodized Titanium Plate Immersed in Simulated Body Fluid

**DOI:** 10.1155/2019/7826373

**Published:** 2019-07-01

**Authors:** Ryoji Sawada, Yuya Katou, Hirofumi Shibata, Max Katayama, Toru Nonami

**Affiliations:** ^1^Faculty of Engineering, Chukyo University, Showa-ku Yagotohonmachi 101-2, Nagoya 466-8666, Japan; ^2^Graduate School of Engineering, Chukyo University, Showa-ku Yagotohonmachi, Nagoya 466-8666, Japan

## Abstract

Titanium-based materials are widely used for implant treatments such as artificial dental roots. Surface treatment has the potential to improve not only the biocompatibility but also the chemical and mechanical durability of the surface without changing the mechanical properties of the metal. A relatively thick titanium oxide film can be formed by the anodic oxidation method. Phosphoric acid or sulfuric acid electrolytic solution has previously been used for anodic oxidation. Such anodized films have excellent film hardness, abrasion resistance, and adhesion. In this study, titanium plate was anodized using an aqueous solution of sulfuric acid in which titanium oxide powder was suspended. A 2800-nm-thick titanium oxide film was formed, which was thicker than that obtained using phosphoric acid electrolyte. The titanium plate was immersed in simulated body fluid for 1 day to evaluate the photocatalytic activity and protein adsorption ability, and a homogeneous crack-free hydroxyapatite layer was formed. This titanium plate showed high methylene blue bleaching capacity. The adsorption ability of the acidic protein of the anodized titanium plate subjected to the above treatment was high. This suggests that this titanium plate has antimicrobial properties and protein adsorption ability. Thus, we report that a titanium plate, anodized with a sulfuric acid aqueous electrolyte solution containing suspended TiO_2_ powder and immersed in simulated body fluid, might behave as an antibacterial and highly biocompatible implant material.

## 1. Introduction

At present, titanium type materials such as titanium and titanium alloy are widely used for implant treatments such as artificial dental roots [1]. Osseointegration was discovered in 1952 by Branemark et al., who indicated that titanium-based material is effective as an implant material [2].

Titanium-based materials have high chemical activity, and their surfaces react with moisture in the air to form a natural film of titanium oxide [3, 4]. This oxide film leads to good biocompatibility [5]. Furthermore, products made of titanium-based materials have many merits such as being light weight, strong, and corrosion resistant. However, in implant treatments, relatively long periods of time are required until the titanium-based material is fixed to the bone tissue, and infections due to implants called peri-implantitis are a problem [6]. In order to avoid such risks, implant materials that are highly antimicrobial and easily bond to bones are required.

Surface treatment has the possibility of improving not only biocompatibility but also chemical and mechanical durability of the surface without changing the mechanical properties of the metal. There are various methods for surface treatment of titanium-based materials, such as plasma spraying [7], acid etching [8], sandblasting, anodic oxidation [9, 10], titanium powder coating by plasma spraying [10], and hydroxyapatite thin film formation [11–13].

A relatively thick titanium oxide film can be formed by anodic oxidation using a phosphate or sulfuric acid electrolyte solution. Such anodized films have excellent film hardness, abrasion resistance, and adhesion [14].

Miyazaki et al. [15] reported anodic oxidation using a mixed solution of phosphoric acid and hydrogen peroxide, resulting in an oxide film with high degree of adhesion. It is also known that titanium oxide film changes from an amorphous state to an anatase type or a rutile type due to an increase in applied voltage during anodic oxidation [16].

Titanium oxide exhibits photocatalytic reaction when absorbing light of a specific wavelength [17, 18]. The active enzyme generated at this time has the ability to decompose the surrounding organic matter. Therefore, there is a possibility that antimicrobial properties can be imparted by anodizing the titanium material for implantation and forming a titanium oxide film having photocatalytic activity on the surface layer [19]. Masahashi et al. [20] have investigated the photoexcitation function of a titanium oxide film fabricated by anodic oxidation method with an aqueous solution of sulfuric acid and report that it has photocatalytic activity.

Regarding the biocompatibility of biomaterials, it is suggested that proteins adsorbed at the initial stage of the implant may influence subsequent cell responsiveness and as a result determine osteoconductivity of the material. The role of albumin, the most abundant protein in the circulatory system, has been studied as the trigger protein of osteoconduction [21]. It is suggested that adsorption properties of albumin may influence osteoconductivity of biomaterials [22–25].

We previously reported that when titanium plate is anodized in electrolyte solution containing ceramic powder such as titanium oxide suspended in 5% sulfuric acid aqueous solution, the oxide film becomes thick [26]. Furthermore, when titanium oxide film is immersed in simulated body fluid (SBF) [27], it has apatite (HAp) forming ability [26].

Anodized titanium plate has surface roughness that is known to have a positive influence on the improvement of affinity with the living body. Furthermore, it is reported that the photocatalytic activity of an oxide thin film is improved by increasing the film thickness or making it porous [28, 29].

Therefore, this study attempted to develop a highly biocompatible titanium material. Titanium plate anodized by using a sulfuric acid electrolyte solution in which titanium oxide powder was suspended was immersed in SBF to evaluate photocatalytic performance and protein adsorption ability. In order to precipitate a homogeneous HAp membrane in short time period, a pseudo body fluid with a phosphorus concentration of 3.83 times the pseudo body fluid used in the previous report was used [26]. Decoloring performance of methylene blue (hereinafter abbreviated as Mb) was evaluated to study photocatalytic activity. Adsorption capacity of bovine serum albumin (hereinafter referred to as BSA) was examined to evaluate protein adsorption ability. As a comparative material, we used a sample anodized by using phosphoric acid electrolyte that has been previously studied.

## 2. Materials and Methods

### 2.1. Anodic Oxidation of Titanium Plate

Titanium plate (pure titanium, JIS H-4600, 10 mm × 10 mm × 1 mm) was washed with 10% aqueous hydrogen peroxide for 10 min (hereinafter referred to as STD-Ti). The titanium plate was anodized with direct current (3 A, 160 V) for 10 min, under the following conditions. A titanium plate was used for the cathode. The three electrolytic solutions used comprised: (1) aqueous solution of 5% sulfuric acid and 10% phosphoric acid; (2) aqueous solution of 5% sulfuric acid; and (3) 0.01 g of titanium oxide powder (MT-150A; Tayca Corporation) in 200 ml of 5% sulfuric acid aqueous solution, as per our previous study [26]. The hydrogen peroxide and electrolyte were special grade reagents from Wako Pure Chemical Industries, Ltd.

### 2.2. Immersion of Titanium Plate in SBF

The titanium plate was immersed in 500 mL of a SBF (special grade reagent, Wako Pure Chemical Industries, Ltd.) having the composition shown in Table 1 and stirred for 1 h in a water bath set at 40°C (TBS451PB, ADVANTEC), then left to stand for 1 day. Thereafter, the titanium plate was taken out and dried in a constant temperature oven.  Table 2 shows a list of abbreviations of the names of the prepared samples.

### 2.3. Surface Observation

Surface observation of the sample was performed at an accelerating voltage of 10.0 to 30.0 kV using a scanning electron microscope (SEM: S-2600N, HITACHI; JSM-IT100, JEOL). An energy dispersive X-ray analyzer (EDS) was added to a scanning electron microscope (JSM-IT100, JEOL) to perform elemental analysis on the surface of the titanium plate.

### 2.4. Auger Analysis

An Auger electron spectrometer (JAMP-9500F, JEOL) was used to perform Auger electron spectrometry (AES) measurements and depth direction analysis of the sample surface. Measurement conditions were primary electron beam: 10 keV, 2.0 × 10^−8^ A; beam diameter: 30 m; sputtering conditions were Ar, 3.0 keV, 30 nm/min (SiO_2_ conversion).

### 2.5. Quantitative Analysis with Electronic Probe Microanalyzer

After embedding the sample in epoxy resin, it was mirror polished, and surface analysis of sample cross section and backscattered electron image (BEI) were observed with an electron probe microanalyzer (EPMA: JXA 8530 F, Nippon Denshi), at a pressurized voltage of 15 kV and an irradiation current of 4.0 × 10^−8^ A.

### 2.6. Powder X-Ray Diffraction

Samples were identified and analyzed by powder X-ray diffraction (XRD: MiniFlex; Rigaku Corporation). Cu was used for the X-ray tube. For the filter, Ni filter was used to remove K*β* ray.

### 2.7. Decolorization of Mb Aqueous Solution

Each sample was immersed in an aqueous solution of Mb (3 ml, 10 ppm) in a spectrophotometer cell. The cells were initially placed in the dark for 30 min and then irradiated for 60 min using UV light (27 W, Sankyo Denki). The light intensity of wavelengths of 380 nm or less of the UV light was 6200 *μ*W/cm^2^. Absorbance of Mb aqueous solution at a wavelength of 660 nm was measured with a spectrophotometer (U-5100, HITACHI) every 10 min, and the concentration of Mb was determined from the calibration curve.

### 2.8. Protein Adsorption

In order to evaluate protein adsorption ability, each titanium plate was placed in 4 ml BSA solution of 0.100 g/L. The room temperature was set at 23°C. Stirring was carried out for 24, 48, and 72 h in a rotary incubator. Thereafter, the absorbance at 280 nm was measured with a spectrophotometer, and the concentration of BSA was obtained from the calibration curve.

## 3. Results

### 3.1. Observation Result of Titanium Plate

From the appearance of the titanium plate after anodic oxidation, the surface of Sp-Ti was darker in color than S-Ti. Further, residual powder of titanium oxide or the like could not be observed on the surface of Sp-Ti. There was no apparent change in the electrolytic solution after anodization.

**Figure 1:**
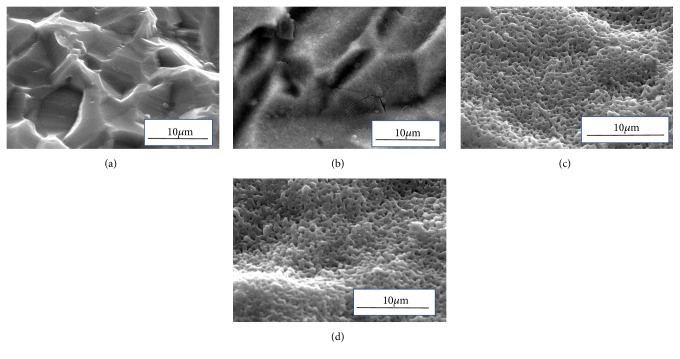
SEM photos of the surface of the Ti plate samples. (a) STD-Ti, (b) P-Ti, (c) S-Ti, and (d) Sp-Ti.

Figure 1 shows an SEM photo of the titanium plate samples. There was no undulation on the surface of STD-Ti and no pores were observed. The surface of the P-Ti is more undulating than the STD-Ti. S-Ti and Sp-Ti showed remarkable formation of pores that were not found in STD-Ti and P-Ti. Sp-Ti developed numerous submicron-sized pores as compared to the other titanium plates. These holes have a unique morphology in which the surroundings are raised. The pore size is widely distributed up to a maximum of 1-*μ*m diameter [27].

**Figure 2:**
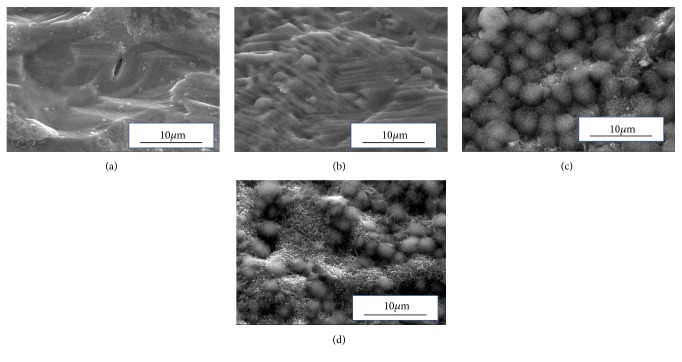
SEM photos of the surface of the samples after immersion in SBF for 1 day. (a) SBF-STD-Ti, (b) SBF-P-Ti, (c) SBF-S-Ti, and (d) SBF-Sp-Ti.

**Figure 3:**
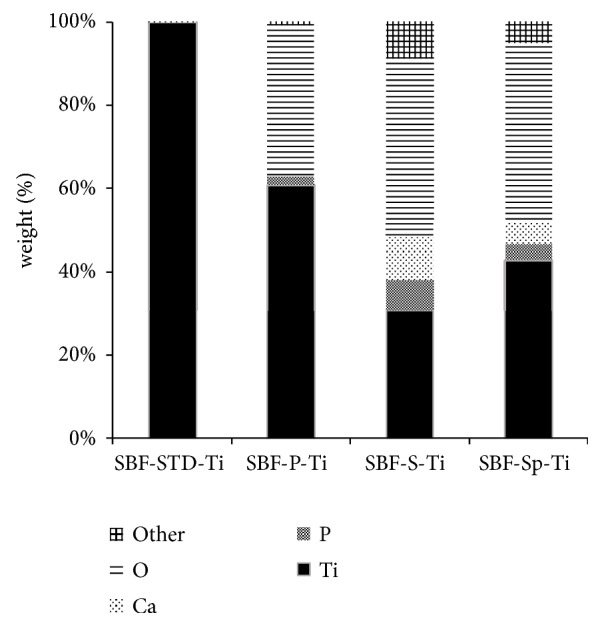
EDS results on the surface of the Ti plates after immersion in SBF for 1 day.

Figure 2 shows an SEM photo of the samples after immersion in SBF for 1 day. For SBF-S-Ti and SBF-Sp-Ti, many new rounded particles were confirmed on the titanium plate surface.  Figure 3 shows the results of elemental analysis by EDS. The presence of calcium could not be detected on SBF-STD-Ti and SBF-P-Ti. On the other hand, calcium was detected on SBF-S-Ti and SBF-Sp-Ti.

### 3.2. Auger Analysis Result

**Figure 4:**
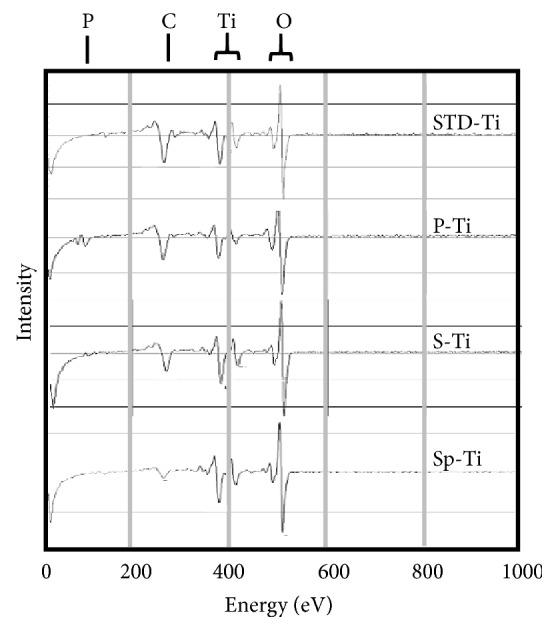
AES spectra of the outermost surface of each Ti plate.

Figure 4 shows the outermost surface AES spectrum of each sample. Titanium, oxygen, and carbon were mainly detected in any of the samples. In P-Ti, a trace amount of phosphorus was observed. A very small amount of sulfur was detected in S-Ti and Sp-Ti.

**Figure 5:**
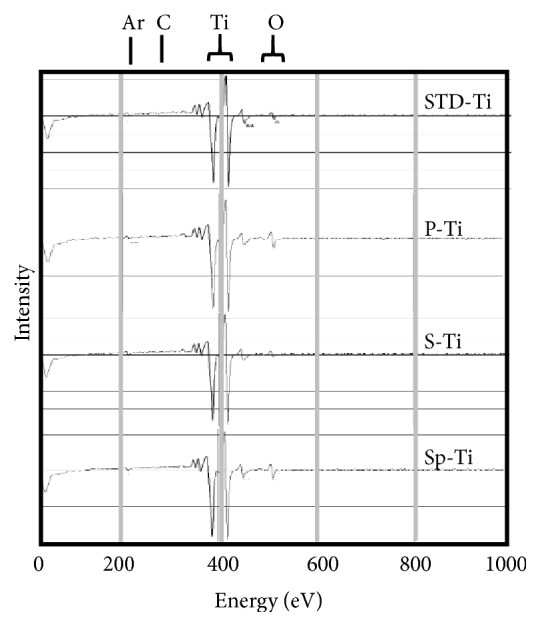
AES spectra of bottom layer after depth direction analysis of each Ti plate.

For the AES spectrum of the lowest layer after analysis in the depth direction shown in Figure 5, titanium was detected in all samples. There was an extremely small amount of oxygen and argon used for sputter etching.

### 3.3. Quantitative Analysis Results with Electronic Probe Microanalyzer

**Figure 6:**
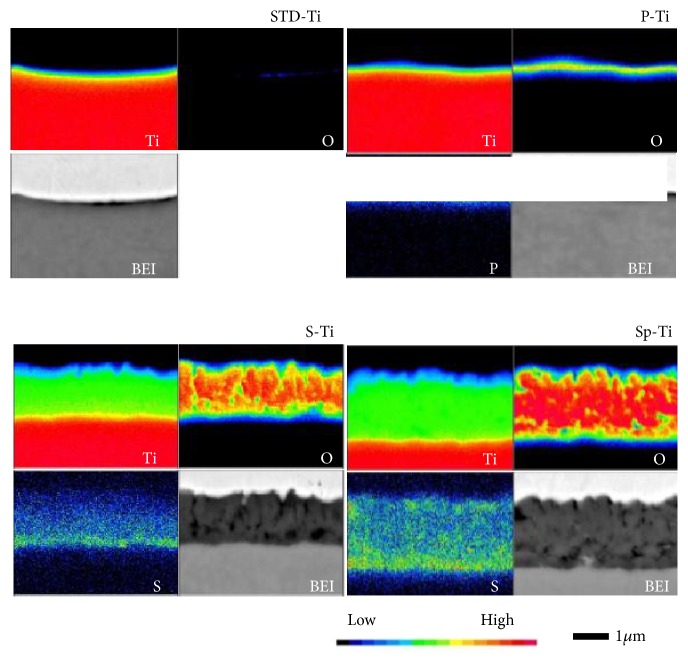
EPMA analysis of cross sections of the anodized Ti plates.

The surface analysis result and the BEI of the cross section EPMA are shown in Figure 6. Based on these results, the thickness of the oxide film of each sample was about 300 nm for P-Ti, about 1800 nm for S-Ti, and about 2800 nm for Sp-Ti.

### 3.4. XRD Analysis

**Figure 7:**
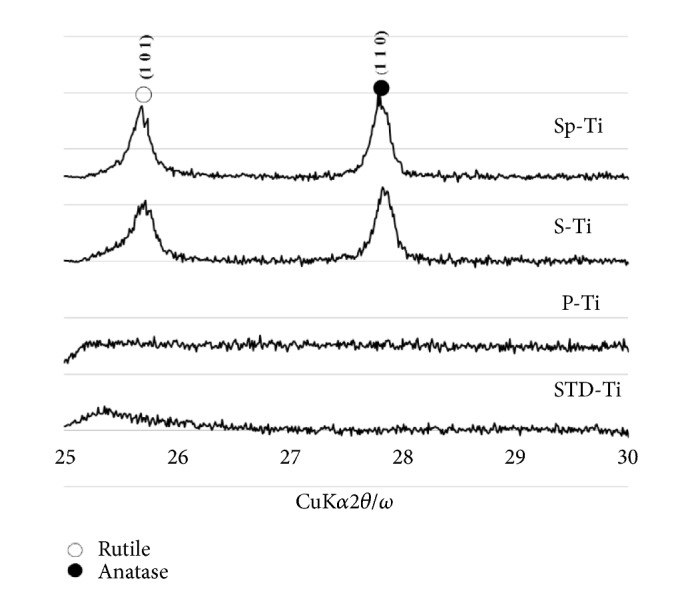
XRD patterns of Ti plate samples.

**Figure 8:**
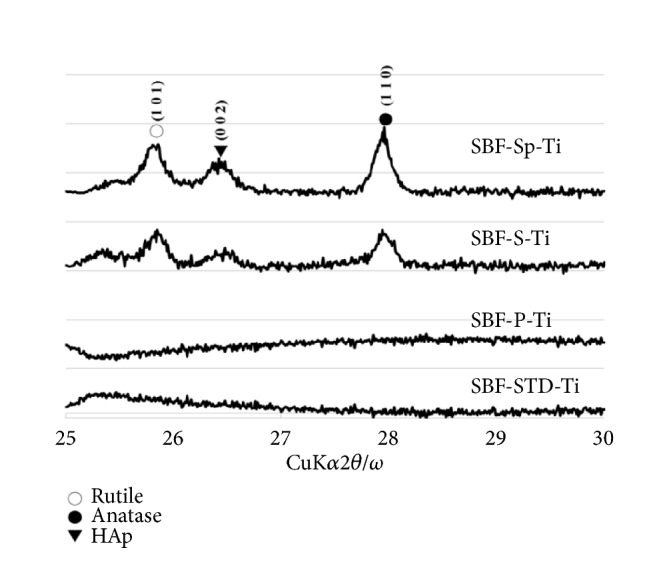
XRD patterns of Ti samples after immersion in SBF for 1 day.

Figure 7 shows the XRD pattern of the titanium plate samples. There were no diffraction peaks of titanium oxide in STD-Ti and P-Ti; however, diffraction peaks of anatase and rutile were confirmed in S-Ti and Sp-Ti.  Figure 8 shows the XRD pattern of the surface of the titanium plate after immersion in the SBF for 1 day. As shown in Figure 8, SBF-S-Ti and SBF-Sp-Ti have diffraction peaks of HAp at around 26° in addition to the peaks of anatase and rutile. No diffraction pattern of octacalcium phosphate (OCP) at around 4.7° was observed.

### 3.5. Mb Decolorization Result

**Figure 9:**
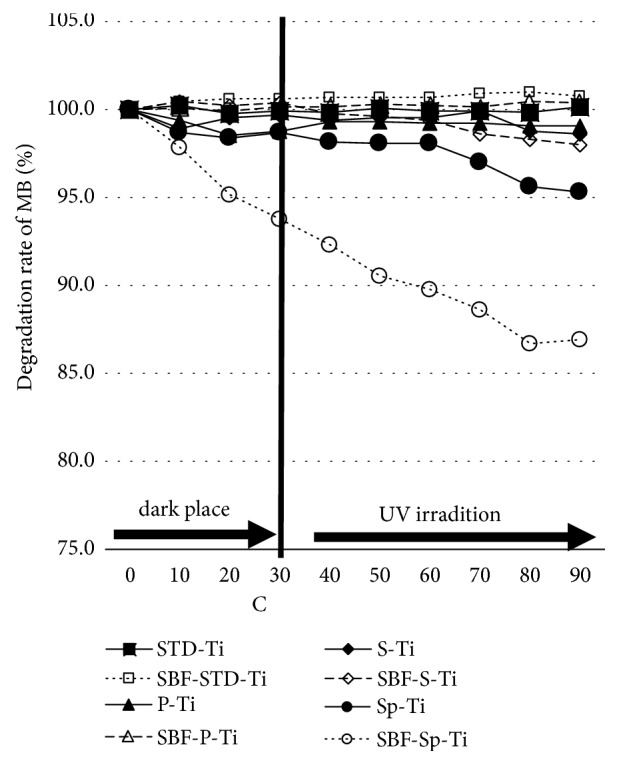
Changes in concentration of Mb in aqueous solution with immersion of the Ti samples, with and without UV light.

Figure 9 shows the results of the Mb decolorization experiment. After 30 min in the absence of light, the rate of decrease of Mb concentration was the highest for SBF-Sp-Ti, but barely changed for STD-Ti, P-Ti, SBF-STD-Ti, and SBF-P-Ti. The concentration of the Mb aqueous solution 60 min after UV irradiation was the lowest for SBF-Sp-Ti at 87%. On the other hand, it was around 95% in Sp-Ti, whilst STD-Ti, P-Ti, SBF-STD-Ti, and SBF-P-Ti showed almost no decrease.

### 3.6. BSA Adsorption Result

**Figure 10:**
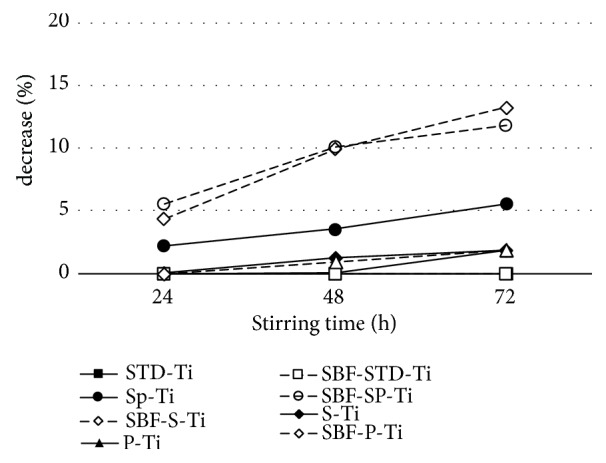
Changes in concentration of BSA when immersing Ti samples in BSA aqueous solution.

The results of the BSA adsorption experiment are shown in Figure 10. The BSA concentration decreased by 5.57% for Sp-Ti, which was the highest of the samples only subject to anodic oxidation. For samples immersed in SBF, BSA concentration decreased by about 13% for SBF-S-Ti and about 11% for SBF-Sp-Ti.

## 4. Discussion

An oxidation layer with a thicker film thickness (2800 nm) could be obtained by anodizing a titanium plate with a sulfuric acid electrolyte solution in which titanium oxide powder was suspended. This agrees with our previous report [26]. This can be seen from the results shown in Figures 1–6 and the fact that the color was darkened by visual observation and the like.

Figure 2 shows that immersion in the SBF, SBF-S-Ti, and SBF-Sp-Ti showed deposits of new material that covered the titanium plate.  Figure 3 shows that both P and Ca were detected on the surface of these samples.

Furthermore, the XRD results in Figures 7 and 8 show diffraction peaks that are estimated to be HAp were confirmed in SBF-S-Ti and SBF-Sp-Ti. From the above, it is assumed that HAp is precipitated by immersing SBF-S-Ti and SBF-Sp-Ti in SBF. It can be considered that the film thickness of the oxide layer is large and because S-Ti and Sp-Ti have many pores, they are in a situation where HAp is more likely to precipitate HAp than for other samples.

In our previous report [26], there was no new product formed on the surface after 3 days of immersion in SBF. However, in this study, HAp was precipitated after only one day. In this study, in order to precipitate HAp at an early stage, the concentration of phosphorus in the SBF used was set to 3.83 times the value used in [26].

In the previous report, many cracks of the HAp layer were observed 7 days after dipping in the SBF, but were not found in this study. Usually in the SBF, first, an OCP layer forms that subsequently becomes HAp. Therefore, it is reported that volume change occurs and cracks are generated in that layer [26]. In this study, since the SBF composition used is different, an OCP layer with lower density was generated. Therefore, cracks did not occur even if volume change occurred during HAp conversion.

As shown in Figure 9, the concentration of Mb after 30 min in the dark decreased the most when SBF-Sp-Ti was immersed. As shown in Figure 8, SBF-Sp-Ti has the largest diffraction intensity of HAp. Therefore, it is considered that HAp on the SBF-Sp-Ti surface adsorbs Mb.

The Mb concentration was the lowest at 60 min after UV irradiation when SBF-Sp-Ti was immersed. SBF-Sp-Ti is considered to have high photocatalytic activity because it has a large number of pores in the titanium dioxide film and has a large film thickness.

Funakoshi et al. [30] reported that when a composite material coated with HAp crystals on the surface of TiO_2_ particles is irradiated with UV, the HAp holds electrons generated on the surface of the TiO_2_. This limits recombination with holes and photocatalyst. Thus, the activity improved.

Therefore, the same effect was also obtained in this study, which would suggest that SBF-Sp-Ti has high photocatalytic activity.

As shown in Figure 10, when SBF-S-Ti and SBF-Sp-Ti were immersed in the BSA aqueous solution, the BSA concentration decreased by a higher degree than when the other titanium samples were immersed. We think that the decrease in BSA concentration by SBF-S-Ti and SBF-Sp-T is mainly due to adsorption by HAp. According to Kawasaki et al., acidic proteins such as BSA are adsorbed by Ca^2+^ sites of HAp [31, 32].

## 5. Conclusions

In this study, titanium plate was anodized using an electrolyte of sulfuric acid aqueous solution in which titanium oxide powder was suspended, and further immersed in simulated body fluid (SBF) to evaluate photocatalytic activity and protein adsorption ability.

(1) A 2800-nm-thick titanium oxide film was formed on the titanium plate after anodic oxidation. After dipping in the SBF, a homogeneous crack-free hydroxyapatite (HAp) layer was formed.

(2) SBF-Sp-Ti showed high Mb bleaching ability. From this, it was proposed that the titanium plate subjected to anodic oxidation with HAp precipitated on the surface had antimicrobial properties.

(3) The adsorption ability of acidic protein of SBF-Sp-Ti plate was improved.

The results indicate that a titanium plate dipped in SBF after anodic oxidation can be expected to be an implant material with antimicrobial properties and high biocompatibility.

## Figures and Tables

**Table 1:** Inorganic ions concentration in SBF.

Ion	mM	Ion	mM
Na^+^	30.6	Cl^−^	28.1
K^+^	0.831	HPO_4_^2−^	1.62
Ca^2+^	0.45	H_2_PO_4_^−^	0.294

**Table 2:** Sample names of the prepared Ti plates.

The electrolytic solution used for anodizing	Sample name	
	After anodizing	After simulated body fluid immersion
Non-processing	STD-Ti	SBF-STD-Ti
Phosphoric acid	P-Ti	SBF-P-Ti
Sulfuric acid	S-Ti	SBF-S-Ti
Sulfuric acid + TiO_2_ powder	Sp-Ti	SBF-Sp-Ti

## Data Availability

The data used to support the findings of this study are included within the article.
